# Validating a 5G-Enabled Neutral Host Framework in City-Wide Deployments

**DOI:** 10.3390/s21238103

**Published:** 2021-12-03

**Authors:** Adriana Fernández-Fernández, Carlos Colman-Meixner, Leonardo Ochoa-Aday, August Betzler, Hamzeh Khalili, Muhammad Shuaib Siddiqui, Gino Carrozzo, Sergi Figuerola, Reza Nejabati, Dimitra Simeonidou

**Affiliations:** 1 i2CAT Foundation, 08034 Barcelona, Spain; lochoa@redhat.com (L.O.-A.); august.betzler@i2cat.net (A.B.); khalili.hamzeh@gmail.com (H.K.); shuaib.siddiqui@i2cat.net (M.S.S.); sergi.figuerola@i2cat.net (S.F.); 2 High Performance Networks Group, Smart Internet Laboratory, Faculty of Engineering, University of Bristol, Bristol BS8 1QU, UK; carlos.colmanmeixner@bristol.ac.uk (C.C.-M.); Reza.Nejabati@bristol.ac.uk (R.N.); dimitra.simeonidou@bristol.ac.uk (D.S.); 3 Nextworks s.r.l., 56122 Pisa, Italy; g.carrozzo@nextworks.it

**Keywords:** 5G, NFV, neutral host, network slicing, city-wide deployments, testbed design

## Abstract

Along with the adoption of 5G, the development of neutral host solutions provides a unique opportunity for mobile networks operators to accommodate the needs of emerging use-cases and in the consolidation of new business models. By exploiting the concept of network slicing, as one key enabler in the transition to 5G, infrastructure and service providers can logically split a shared physical network into multiple isolated and customized networks to flexibly address the specific demands of those tenant slices. Motivated by this reality, the H2020 5GCity project proposed a novel 5G-enabled neutral host framework for three European cities: Barcelona (ESP), Bristol (UK), and Lucca (IT). This article revises the main achievements and contributions of the 5GCity project, focusing on the deployment and validation of the proposed framework. The developed neutral host framework encompasses two main parts: the infrastructure and the software platform. A detailed description of the framework implementation, in terms of functional capabilities and practical implications of city-wide deployments, is provided in this article. This work also presents the performance evaluation of the proposed solution during the implementation of real vertical use cases. Obtained results validate the feasibility of the neutral host model and the proposed framework to be deployed in city-wide 5G infrastructures.

## 1. Introduction

In the apogee of a new digital era, communication technologies and businesses evolved towards 11 billion interconnected devices worldwide, triggering a rapid adoption of online and mobile digital services [1,2]. This reality of continuously increasing traffic poses new challenges in terms of performance and business sustainability. In this context, the fifth-generation (5G) of mobile networks is a promising solution for the needs of an extremely mobile and hyper-connected society [3].

The 5G architecture combines emerging Radio Access Network (RAN) technologies with advances in Software-Defined Networks (SDN) and Network Function Virtualization (NFV) [4]. Such enabling technologies unleash the potential of 5G in terms of service orchestration, infrastructure virtualization, cloud and edge computing, end-to-end network slicing, and mobile communication with higher throughput and lower latency. In this way, 5G copes with performance challenges by supporting a much larger and diverse number of services, including data-intensive and delay-sensitive applications (e.g., immersive reality, industry 4.0, smart city) [5]. However, 5G still faces challenges in the integration and deployment of such enablers, as well as in the evaluation of business feasibility and sustainability.

Enabled by the introduction of network slicing in 5G [6], the neutral host model is changing the whole telecommunication business ecosystem by transforming the traditional market to be more pervasive and open to new opportunities for infrastructure and service providers [7]. Under the neutral host model, infrastructure and service providers can find new ways to monetize their services and share the cost of infrastructure upgrade (capital expenditure (CAPEX)) and ensure a quick return of investments (ROI) and business sustainability [8].

Several private and public initiatives are supporting research and innovation projects to deal with challenges for 5G technology realization (5G Public Private Partnership (5G PPP) in Europe [9] and the International Mobile Telecommunication 2020 (IMT2020) at International Telecommunication Union (ITU) [10]. However, few projects in the ecosystem are focused on infrastructure sharing and business changes to improve, integrate, and demonstrate 5G neutral hosting capabilities in a hyper-connected city infrastructure with real use cases. Hence, as part of 5GPPP and European Commission (EC) Horizon 2020 (H2020) initiatives, the 5GCity project [11] focused on the design, development, deployment, and validation of a novel 5G-enabled neutral host framework for real-world deployments in three European cities: Barcelona (ESP), Bristol (UK), and Lucca (IT).

In this article, we refer to the term 5G-enabled neutral host framework to define a system capable of dynamically managing and orchestrating a virtualized network infrastructure to allocate 5G services with disparate requirements for multiple relevant stakeholders. The 5G-enabled neutral host framework provides multitenancy to a city-wide deployment by realizing and combining enhancements of 5G enabling technologies required by the neutral host model. This article revises the main achievements and contributions of the 5GCity project, focusing on the deployment and validation of the proposed 5G-enabled neutral host framework in the three aforementioned cities. The developed neutral host framework consists of a software platform for slicing and orchestrating computing and network resources from a 5G-enabled cloud, edge, and radio infrastructure. A detailed description of the framework implementation, in terms of functional capabilities and practical implications in city-wide deployments, is described in this work. In addition, this paper presents the validation of the proposed neutral host framework in city-wide deployments and provides the obtained results of measuring several Key Performance Indicators (KPIs) with real smart-city and media use cases.

The remainder of this article is organized as follows. Section 2 provides some background related to the neutral host model and enabling technologies for network slicing such as cloud/edge orchestration and RAN virtualization. Section 3 details the overall framework design of the proposed neutral host platform and architecture. Then, in Section 4, we describe the city-wide deployment of the proposed framework for each one of three considered city pilots (Barcelona, Bristol, and Lucca). Section 5 outlines the envisioned workflow for use case deployments using the developed neutral host platform. Following that, the framework validation and obtained results in terms of several KPIs are discussed in Section 6. Finally, Section 7 concludes this article.

## 2. Background on Neutral Host Concept and Enabling Technologies

The 5G technology is increasing network convergence, flexibility, and mobile broadband capacity in response to the growth in number and diversity of consumers, industries, and service demands in society [2,12]. However, without network infrastructure sharing [13,14], deployments of 5G networks in dense environments will not be feasible and sustainable because it will require hundreds of isolated access networks, which is unrealistic.

A neutral and sliceable 5G infrastructure is a promising solution for network infrastructure sharing challenges [10,15]. From a user’s point of view, the system behavior and services using the resources of a neutral host should be available without user intervention and, ideally, these should be seamless and identical to those provided by their hosted clients’ dedicated resources. This concept was around for some time, but within 5G ecosystems it cannot exploit its full potential due to:

- An increasing need for enhanced and ubiquitous connectivity in urban context coupled with more demanding requirements of radio coverage and bandwidth.
- The pivotal role within 5G of smart cities, in which municipalities may act as potential 5G neutral host providers.
- A neutral host framework is a perfect candidate to fully satisfy the 5G requirements for different use cases (e.g., eMBB, URLLC, mMTC) concurrently deployed over a shared infrastructure.

The neutral host model has an important business dimension focused on the creation of new Service Level Agreements (SLAs) categories to rule the interactions between the host and content/service providers [16,17]. A key enabler of the neutral host model is a flexible and automated network slice allocation allowing programmability of policies according to the created SLAs, while enforcing dynamic up/down scaling decisions of infrastructure resources, assigned to service providers. As such, a tenant uses the neutral host framework to establish end-to-end segmented slices, representing partitions of the network, storage, and computing resources. In turn, those slices are leased to service providers to operate allocated resources for the mapping of their services.

Indeed, a neutral host framework provides automated or dynamic multi-tenancy by combining a wide range of well-known technical enablers via end-to-end network slicing. While network slicing is not new in the academia and industry community, the 5G technology and the neutral host model are revitalizing the interest from the community by extending it to the next level [18].

The Next Generation Mobile Networks (NGMN) [15] redefines the classical network slicing of a single virtual network (e.g., Layer 2 (L2) Virtual Local Area Networks (VLANs) [19], Layer 2 and Layer 3 (L2–L3) Virtual Private Networks (VPNs) [20]) to an end-to-end network slicing formed by several virtual networks mapped across cloud, edge, and RAN infrastructures. Later, the 3rd Generation Partnership Project (3GPP) with its standard [21] and the European Telecommunications Standards Institute (ETSI) NFV division with its standard [16] extended the NGMN definition of network slicing as a set of Network Services (NSs) interconnected by VLANs representing slices (or resource segments) from multiple infrastructures.

Overall, end-to-end network slicing in 5G requires an NFV Management and Orchestration (MANO) solution to deploy Virtual Network Functions (VNFs) on cloud/edge resources and a virtualized RAN solution to slice and control the radio resources [22].

### 2.1. Cloud/Edge Computing and Orchestration

A Multi-Access Edge Computing (MEC) network architecture uses edge resources to enable cloud computing capabilities and IT service environments at the edge of the cellular network [23]. As a result, this environment is characterized by applications running close to the User Equipment (UE).

The neutral host model leverages multiple MEC deployments to fully cope with 5G requirements in terms of bandwidth, coverage, and latency. Indeed, distributed edge resources allow neutral host frameworks to deploy end-to-end services across distributed pools of edge resources, enforcing ultra-low latency and high bandwidth, while real-time access to radio network information can be leveraged by applications.

A MEC architecture poses some real challenges for the design of end-to-end services, mainly since the resources locally offered can be limited, thus highlighting the need for tightly centralized orchestration, which can dynamically operate the life-cycle of edge computing applications [17,24].

End-to-end network slicing, over the distributed cloud and edge resources for enhanced computing and processing capabilities, requires extensions or a new NFV MANO framework to support multitier orchestration, covering also different edges (e.g., extended edge). As such, multitier orchestration in network slicing is getting attention from industry and academia [23].

Among well-known NFV MANO solutions only Open Source MANO (OSM) [25], an ETSI-compliant and hosted project, and SONATA [26] are capable of network slicing lifecycle management. As a result, our platform leverages ETSI OSM as the NFV MANO solution [27]. Moreover, to provide pervasive neutral host services our framework goes beyond the edge to the extended edge by deploying a multitier NFV Orchestrator (NFVO) module [28,29] (cf. Section 3).

### 2.2. Virtualized Multiradio Access Network

The Cloud Radio Access Network (C-RAN) concept [30] defines the splitting and virtualization of cellular building blocks by placing Layer 1 (L1) functions close to the antennas and virtualized functions from upper layers integrated with NFV and SDN (i.e., Software Defined Radio (SDR)) architectures. In this context, projects such as OpenRAN [31], O-RAN Alliance [32], and NG-RAN architecture [33] are focusing on the splitting and virtualization of radio functions to integrate with SDN and NFV platforms for slicing.

Targeted for 5G framework and strictly coupled with edge architectures, the virtualized RAN (vRAN) approach empowers the neutral host scenario with the capability of sharing the radio access part, by slicing its resources in multiple tenants, each one operated by a different Mobile Virtual Network Operator (MVNO). Moreover, given the high flexibility of the neutral host framework, different sharing and architecture models (i.e., Multioperator Radio Access Network (MORAN) and Multioperator Core Network (MOCN)) can be realized, thus providing a wide range of deployment solutions.

For neutral host and 5G deployments, network slicing between Multiradio Access Technologies (Multi-RAT) will be essential. Hence, efforts from industry and academia are focusing on the integration of Long Term Evolution (LTE) and 5G New Radio (5GNR) technologies with various slicing approaches [34] (e.g., assigning shares of the available airtime to different clients) from different wireless technologies (e.g., Li-Fi, Wi-Fi 6).

To integrate multiple RAN technologies in the envisioned network slicing, a RAN controller is essential to slice, allocate, and monitor radio resources (in similar ways that the Virtual Infrastructure Manager (VIM) does with computing resources). A recently proposed SODALITE framework integrates back-haul traffic from Wi-Fi and 4G/5G in dense small cell networks [35]. Then, two well-known RAN controller platforms are the flexRAN [36] built on open-source LTE stack with OpenAirInterface [37] and EmPOWER [38] which adds Wi-Fi and Long Range (LoRa) radio technologies to LTE. Our neutral host framework goes beyond a novel RAN controller supporting LTE and Wi-Fi by adding capabilities to multiple RAN controllers from multiple vendors and radio technologies [39] (cf. Section 3).

### 2.3. Our Contribution

Some other relevant 5GPPP projects that also deal with expanding network slicing functionalities are 5G SESAME [40], 5G ESSENCE [41], and SLICENET [42]. In particular, 5G SESAME and 5G ESSENCE focused more on the integration of RAN virtualization and slicing with SDN and NFVO architectures, while SLICENET focused on a platform for E2E network slicing beyond 5G technology. Compared to the aforementioned approaches, our neutral host framework innovates in the integration and extension of 5G enablers developed by academia and industry to demonstrate network slicing in real city-wide deployments. This materializes with the introduction of novel functional entities to the proposed architecture ( cf. Section 3) (e.g., Multitier Orchestrator; Multi-RAT RAN Controller) that enhance the system capabilities to better handle multiple technologies for neutral host deployments that meet 5G services requirements.

In addition to the complexity associated with the integration and demonstration of all the technologies mentioned above, implementing a neutral host solution becomes even more challenging when considering real-world city scenarios. In this regard, the infrastructure planning and deployment phases play a fundamental role in achieving the potential added values of the system in operational conditions. While the feasibility of city-driven neutral host deployments was acknowledged from the study of business model perspective in previous works [43,44,45], there is a lack of literature validating the practical implementation of such deployments. The main contributions of this work are precisely oriented to close this gap regarding the deployment and validation of 5G-enabled neutral host frameworks in the real world. To the best of our knowledge, this paper is the first to describe a comprehensive deployment and validation of a 5G-enabled neutral host framework in three different city-wide deployments.

## 3. Overview of Neutral Host Framework

**Figure 1:**
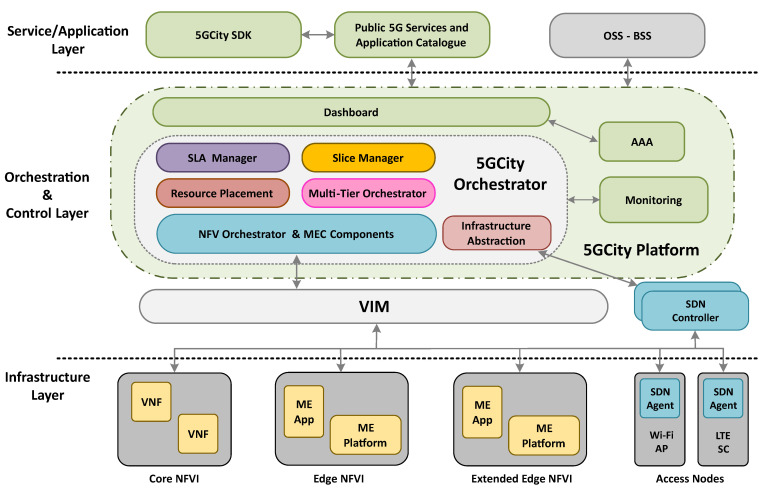
Neutral host framework.

In short, the 5G-enabled neutral host framework allows tenants to create and consume slices using a set of virtualized resources over a common infrastructure. The conceived framework, presented in Figure 1, is split vertically across three layers: Service/Application layer; Orchestration & Control layer; and Infrastructure layer.

### 3.1. Service/Application Layer

In the Application layer, a set of tools oriented to facilitate service design and composition is available for service providers, tenants, and any related third party. In particular, the innovative nature of the neutral host framework is further enhanced at the service layer with a *Software Development Kit* (*SDK*) for network functions developers and service providers to combine new and pre-existing functions for new service deployments. Likewise, a *5G Service & Apps Catalogue* is also provided to store network services previously created and published. This component is also responsible for the onboarding of functions and services into the NFVO.

### 3.2. Orchestration & Control Layer

The Orchestration & Control layer is the logical core of our neutral host framework [27] composed of multiple functional blocks for control, management, and orchestration across its 3-tier architecture. A *Dashboard* with a Graphical User Interface (GUI) and a component for *Authentication, Authorization, and Accounting* (*AAA*) are placed at the northbound side, to facilitate the interaction between infrastructure owners and tenants and to enforce the required security and billing. Network slices of different tenants are properly separated for security reasons, and the full isolation of information and data is preserved.

The *Slice Manager* has a central role in the platform, especially in the provision of the required logic for dynamic creation and management of slices. Each slice is defined as a collection of logical network partitions or chunks, combined with the network services deployed on top of them [46]. Apart from managing the registration of infrastructure resources and the creation and removal of chunks and slices, the *Slice Manager* performs several automated tasks seamlessly, to:

- Activate deployed slices by launching required servers (i.e., mobile core for serving cellular network slices and DHCP servers for IP assignment of Wi-Fi slices), together with the corresponding configuration of radio access chunks.
- Perform required postinstantiation configurations to deploy VNFs, in terms of enabling external connectivity, registering tasks and alerts for monitoring purposes (in the *Monitoring* component), and DNS deployments.
- React to triggered alerts to conduct the corresponding actions (as established by the *SLA Manager* [47]), such as horizontal scaling of specific VNFs.

To compute the optimal allocation of VNFs to be deployed over a given slice, a *Resource Placement* component is also provided. In essence, this component determines the most suitable VNF-to-compute-chunk mapping by taking into consideration the service requirements and the resources usage.

The orchestration capabilities of the presented platform are extended to support NFV/MEC integration following the ETSI MEC specification [24] by complementing the NFVO with MEC components that handle Mobile Edge (ME) applications [28]. In particular, the MEC Application Orchestrator (MEAO) enables the definition and management of ME platforms, applications, and services running on different mobile edge hosts. Likewise, the ME Platform components not only manage the MEC services but also handle the notifications when there are changes in the management of a given ME application or service. Hence, the *Multitier Orchestrator* component [29] is included to provide an abstracted view in front of multiple underlying orchestrators.

Additionally, this platform also enables virtual RAN slicing and RAN function virtualization for 5GNR, LTE, and Wi-Fi. To do so, *SDN-based RAN controllers* are placed beneath the aforementioned components to manage the radio components and enforce many of the required actions. To support multiple underlying RAN controllers and technologies, the *Infrastructure Abstraction* module is located as an intermediate component between the *Slice Manager* and the underlying SDN controllers.

### 3.3. Infrastructure Layer

The Infrastructure layer contains the resources in terms of computing, network, and radio components managed by the neutral host. This layer is graphically divided into several Network Functions Virtualization Infrastructure (NFVI) sections and access nodes to identify the distributed compute and radio architecture conceived for municipalities and infrastructure owners acting as 5G neutral host providers. Moreover, this framework is also aligned with 5G performance requirements by providing edge computing capabilities. This will result in real-time access to radio network information, thereby unlocking the potential of advanced future applications.

## 4. City-Wide Deployments

**Figure 2:**
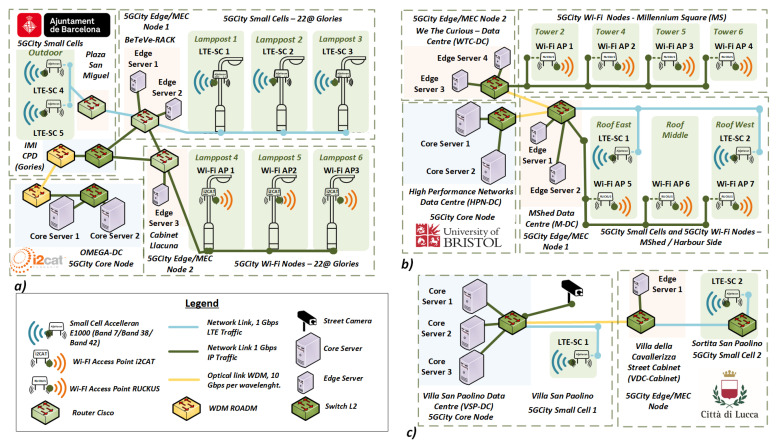
Infrastructure design for (**a**) Barcelona, (**b**) Bristol and (**c**) Lucca.

Following the architecture presented in Section 3, the proposed neutral host framework was deployed in the cities of Barcelona, Bristol, and Lucca. In this section, we describe the deployment of the proposed framework in each one of three considered city pilots. A logical view of the three infrastructure deployments is depicted in Figure 2.

The general methodology followed for deploying the proposed neutral host framework consisted of the following sequential phases in every city pilot:

(i) Infrastructure Deployment: the conceived three-tier architecture, including a RAN tier, an edge tier, which can be further extended to be closer to end-users, and a core Data Center (DC) tier, is mapped into physical infrastructure resources consisting of radio components, edge/MEC servers, and DC servers;
(ii) Infrastructure Setup Validation: to verify the correct installation and performance of the deployed infrastructure in the three cities, a similar set of validation tests was conducted. The main objective of these tests was to verify performance and better profile configurations in the three pilot environments;
(iii) Platform Installation: deployed servers at edge and DC sites in every city provide computing resources to host the different components of the software platform of the neutral host framework. In general, each software module of the platform is installed as a Virtual Machine (VM) in the virtualized computing infrastructure and interconnected to allow the required interaction among them;
(iv) Platform Setup Validation: the validation of the deployed platform consisted of a set of functional tests aimed at verifying the correct integration of the various orchestration elements, as well as the execution of lifecycle management operations for infrastructure resources, slices, and network services.

Next, we first detail the infrastructure components deployed per testbed. Then, we describe the platform implementation, which is a common factor for the three city pilots.

### 4.1. Infrastructure Deployment in the City of Barcelona

**Figure 3:**
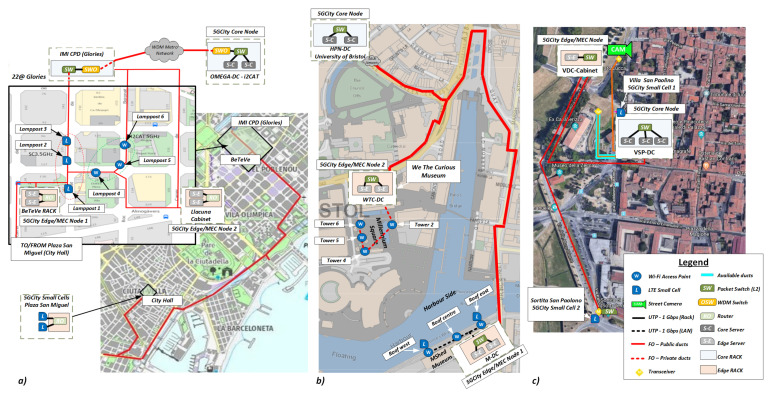
Infrastructure deployed in (**a**) Barcelona, (**b**) Bristol and (**c**) Lucca.

The neutral host infrastructure deployed in Barcelona comprises three city areas: (i) the core node hosted in *OMEGA-DC* at i2CAT Foundation; (ii) the edge computing nodes and on-street RAN deployed in the super square *22@ area (Glòries)*; (iii) an additional RAN deployed within the city hall (*Plaza Sant Miquel*), located in the *Barrio Gòtico* district. This third location added value to the media use cases by providing connectivity in the most central and lively area of the city with the potential to cover major public events. The resulting physical deployment of the neutral host infrastructure interconnecting the three city areas of Barcelona is presented in Figure 3a.

#### 4.1.1. Core Tier

The core DC in Barcelona infrastructure is deployed in the *OMEGA-DC* at i2CAT. This DC hosts two compute servers providing the core NFVI. The fiber infrastructure connects this location to the on-street components deployed in the *22@ area* and the city hall using L2/L3 network devices. To add resilience, two end-to-end fiber connections of 10 Gbps were deployed. Each fiber provides end-to-end redundancy in case of interruptions.

#### 4.1.2. Edge/MEC Tier

The edge/MEC nodes in Barcelona are installed in two locations within the *22@ area*. The primary location is in BeTeVé premises, hosting two edge servers, which act as edge NFVI. The second edge/MEC node is deployed in a street cabinet at the crossing of the *Llacuna* and *Pere IV* streets, where an edge server provides the extended edge NFVI. These two edge locations are equipped with dedicated L2/L3 routers (Cisco ASR920) to connect the edge computing nodes with the core DC located at i2CAT. Likewise, as shown in Figure 3a, the edge/MEC locations are connected to the RAN elements in *22@ area* and the city hall using a dedicated fiber.

#### 4.1.3. RAN Tier

The RAN equipment in the *22@ area* is mounted on six lampposts with their own energy supply and 1 Gbps fiber connection. Three of them are equipped with LTE small cells (Accelleran E1010), whereas the other three lampposts are equipped with Wi-Fi nodes (i2CAT custom hardware). The RAN equipment in the city hall consists of two LTE small cells installed next to the *Salo de Cent* room covering city council meetings and public events. The small cells deployed in Barcelona use Band 42 and follow the TDD config mode 2 on 20 MHz, providing a maximum of 90 Mbps Down-Link (DL) and 10 Mbps Up-Link (UL).

### 4.2. Infrastructure Deployment in the City of Bristol

The neutral host infrastructure deployed in Bristol extends the 5GUK test network [48] by implementing a larger radio coverage and including a new site at the *MShed Museum*. This new location enables a wider experimentation area covering the harbour part on the other side of the *Avon* river. The overall connectivity and main infrastructure locations of the Bristol pilot deployment are depicted in Figure 3b.

#### 4.2.1. Core Tier

The core DC of Bristol infrastructure is deployed at the University of Bristol High-Performance Network Group Data Centre (HPN-DC). More in detail, this DC hosts two compute servers configured as core NFVI, which are interconnected via fiber to the other two pilot locations in *We-The-Curious* (WTC-DC) and *MShed Museum* (M-DC).

#### 4.2.2. Edge/MEC Tier

In Bristol, the edge/MEC nodes are deployed in two locations: the *We-The-Curious* (WTC-DC) and the *MShed Museum* (M-DC). In both locations, standard rack servers and edge-format servers are installed, which are configured to act as edge NFVI. Both locations are interconnected with the core DC and corresponding RAN devices.

#### 4.2.3. RAN Tier

For the RAN in Bristol, a total of four towers in the *Millennium Square* are used, each one equipped with a dedicated Wi-Fi node (Ruckus T710) to provide coverage to the square and the close surrounding areas, down to the harbourside area. Additionally, the *MShed Museum* location hosts two small cells (Accelleran E1000 series) and three Wi-Fi nodes (Ruckus T710) at East Roof, Middle Roof, and West Roof, respectively. The location of each node can be corroborated in Figure 3b. Similar to Barcelona, the small cells deployed in Bristol use Band 42 and follow the TDD config mode 2 on 20 MHz with a maximum throughput of 90 Mbps in DL and 10 Mbps in UL.

### 4.3. Infrastructure Deployment in the City of Lucca

The deployment of the neutral host infrastructure in Lucca was conceived to take into account the very specific historical characteristics of the city (historic walls and pathways on top) and the most appropriate target areas for demonstrations and validations of the use cases planned for Lucca. For this reason, the infrastructure deployment in Lucca has privileged green areas and squares close to the historical wall with pathways, since public events typically occur there. An overview of the physical deployment of the neutral host infrastructure in the Lucca pilot is presented in Figure 3c.

#### 4.3.1. Core Tier

The core DC in Lucca is deployed in *Villa San Paolino* (VSP-DC) hosting three compute servers, one for orchestration and interconnection services (VPN concentrator), whereas the two others act as core NFVI to host the workload from the various use cases. The three servers are interconnected with the edge cabinet and an outdoor CCTV IP camera (required for one of the use cases deployed in this city pilot) via a fiber network. One of the compute servers includes a GPU for video analytic.

#### 4.3.2. Edge/MEC Tier

The edge/MEC node deployed at *Villa della Cavallerizza* is hosted in a street cabinet, which is connected via a fiber link to the core DC in VSP-DC. One compute node is installed in the cabinet, which is used as edge NFVI. In the same cabinet, one L2 switching device interconnects the edge server with the small cell in *Sortita San Paolino* and the core DC.

#### 4.3.3. RAN Tier

In Lucca, the RAN infrastructure is composed of two small cells (Accelleran E1013), which are deployed in two different locations around the city (i.e., *Villa San Paolino* and *Sortita San Paolino*). In this case, the deployed small cells use Band 38 and are configured to follow the TDD config mode 1 on 15 MHz, providing a maximum of 55 Mbps DL and 13 Mbps UL.

### 4.4. Deployment of the Neutral Host Platform

**Figure 4:**
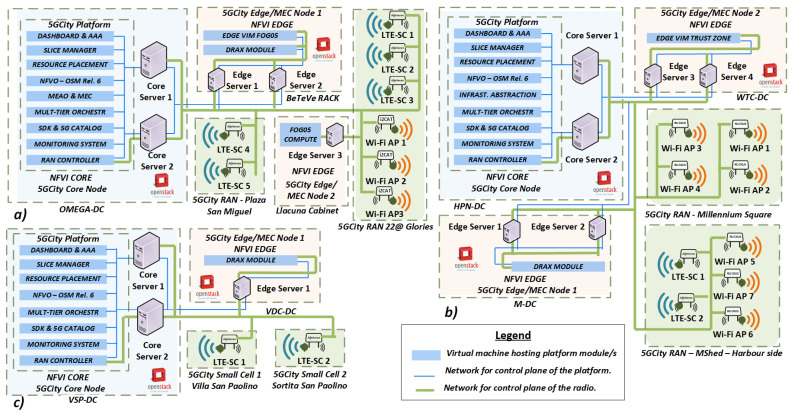
Platform deployed in (**a**) Barcelona, (**b**) Bristol and (**c**) Lucca.

A meticulous planning of computing resources and network connectivity design was required to properly instantiate all the components of the neutral host platform in the three city pilots, following its latest release [49]. For the sake of illustration, Figure 4 represents the mapping of the platform components with respect to the infrastructure deployed in each city. In general, each software module of the platform is deployed as a VM placed in the compute nodes of the core and edge NFVI.

Below, we provide a brief description of the main technologies that enable the deployment of the neutral host platform:

- The VIM was implemented in the core and edge DCs using OpenStack (release Queens). This cloud platform is currently the most widely deployed open-source cloud infrastructure software in the industry.
- Additionally, to support the deployment of NSs based on containers, we also installed Fog05 [50] as the extended edge VIM of the platform. This open-source project enables the deployment of services in resource-constrained devices, which are close to end-users, thus minimizing the service latency.
- To orchestrate the lifecycle of NSs within the 5G-enabled slices, we deployed OSM as the NFVO of the platform.
- Finally, as part of the vRAN capabilities offered by the neutral host framework, we also deployed the dRAX Open Interface RAN Intelligence [51] solution. This cloud-native component runs virtualized in the edge/MEC infrastructure to manage the associated small cells as radio units, which effectively unlocks the potential of 5G network slices for multitenant operators. All this while ensuring low latency and processing at the edge for deployed radio services.

The rest of the components that integrate the neutral host platform (as described in Section 3), were developed as open-source software and released in the GitHub space of the 5GCity project [52].

#### 4.4.1. Automated Deployment

An automated approach was followed to efficiently deploy the platform components in the city-wide pilots. Deployment tasks were divided into two categories, namely Day 0 and Day 1 configurations.

- *Day 0 Configurations:* The tasks automated in this group were related to the creation of VMs for each of the platform components. To this end, we used Terraform [53], a cloud-agnostic management tool that provides a flexible way to define the computing and networking requirements of platform components as a blueprint that can be deployed at any moment.
- *Day 1 Configurations:* Once the VMs are instantiated on the cloud infrastructure, the following task to address is related to the code installation and configuration. This was accomplished using Ansible [54], which has proven to be very efficient to configure, deploy, and orchestrate the code of each platform component.

The aforementioned automated deployment approach allows us to properly replicate the deployment across multiple instances of the platform. In this way, the platform deployment was efficiently conducted saving efforts and time by significantly reducing the probability of error-prone operations.

#### 4.4.2. Platform Deployment Validation

Given the importance of ensuring the proper deployment and functionality of the conceived platform, multiple validation tests were executed. In general, tests were designed taking into account the components involved, the defined interactions, as well as the expected results. Therefore, two groups of tests were conducted:

- *Individual tests:* All elements of the platform were individually tested after accomplishing the deployment of each component to corroborate their functionality. These tests validated the attainment of the expected behavior of every developed module and feature.
- *Integration tests:* To verify the proper interaction between components of the platform, specific integration tests were performed. Particularly, the performance of these tests validated the entire workflow involved in the lifecycle automation of a neutral host framework, in terms of infrastructure (registration, configuration, and removal), slices (creation, activation, and removal), and services (onboarding, instantiation, and removal).

## 5. Use Cases Deployment

The deployment of use cases over the proposed neutral host framework follows a common workflow. In general, tenants access and interact with the framework via the platform Dashboard. The following subsections describe the operations performed by tenants using the platform to deploy different vertical use cases, as described in [55]. For illustrative purposes in this section, we refer to the deployment of a media vertical use case for real-time video acquisition and production at the edge.

### 5.1. VNF and NS Composition and On-Boarding

At first, users create the virtualized functions and services to be instantiated, through the following steps:

- The platform administrator acting as neutral host provider creates a dedicated repository and user account for the media vertical tenant. The referred user is granted the role of Designer, which allows tenants to design functions as well as compose them into services.
- In turn, the media vertical tenant, using the platform SDK, conducts the creation of the required functions and composes an NS for the application.
- Once the service creation is completed, the resulting function and service descriptors are published into the 5G Apps & Services Catalogue of the platform.

### 5.2. Slice Creation and Activation

Next, a customized and dedicated slice is created. In particular, each slice is conceived as a collection of compute, network and radio chunks, as logical and isolated partitions over the common infrastructure. To perform this step, the media vertical tenant composes an end-to-end slice by selecting the desired compute, network and radio infrastructure resources and specifying the requirements to be allocated into the slice. The slice creation request is then processed by the Slice Manager, which interacts with other platform components (i.e., OpenStack, as VIM; OSM, as NFVO; and the RAN Controller, as radio devices manager) to create the required chunks at each network segment.

Following the slice creation, the next step is its activation. Essentially, as the considered media vertical use case requires cellular access for the final users to consume the media application via smartphones attached to the slice, the activation step consists of the instantiation of an open-source mobile core server [56] together with the required configurations of the radio access nodes included in the slice. These configurations include setting the Public Land Mobile Network ID (PLMNID) that is assigned to that slice. Similarly, when Wi-Fi nodes are part of the wireless chunk of the slice, a DHCP server is automatically deployed by the platform as well, to support the service operation in terms of IP addresses allocation.

### 5.3. Network Service Instantiation

Once the function and services descriptors, as well as the slice, are available, the last step is the instantiation of the virtualized service over the given slice. In this step, the application is deployed in the form of VMs or containers. More in detail, the virtualized functions are placed over the compute chunk of the slice and connected to the network chunk, providing end-to-end connectivity with the access chunk of the slice. The successful instantiation of the network services related to the considered media vertical over the neutral host framework are reported by the platform Dashboard and can be also corroborated by checking underlying systems (such as OSM and OpenStack).

Additionally, to complement the programmability principles of the proposed framework, a DNS server is automatically deployed by the platform to support the service operation in terms of IP addresses and domain names resolution.

## 6. Validation of Use Cases

In this section, we focus our attention on the performance validation of the proposed neutral host framework. In particular, several slices are created to demonstrate the multi-tenancy as an intrinsic feature of the neutral host model.

### 6.1. KPIs and Measurement Methodology

The conducted experimental trials enable the measurement of relevant KPIs, which reflect the service requirements and contribute to validating the benefits of the proposed framework. The definition and measurement methodology are described next for each one of the considered KPIs.

#### 6.1.1. User Experienced Data Rate

The minimum data rate required to ensure a sufficient quality experience (without considering broadcast services) [5]. In this evaluation, the measurement of this KPI is done at the application server, by monitoring the throughput achieved by a single UE that generates traffic towards the server.

#### 6.1.2. Data Plane Delay

The time required to transfer a given piece of information between two nodes, measured from the moment it is transmitted by the source to the moment it is successfully received at the destination. This metric was evaluated by computing half of the round trip time experienced between a UE and a remote server.

In particular, in the scope of the proposed framework, this performance was improved by enabling a more suitable allocation of remote servers closer to the end-user, i.e., at the edge to reduce network latency.

#### 6.1.3. Slice Deployment Time (SDT)

The overall time required to deliver an active slice over the neutral host infrastructure. In essence, the SDT refers to the time required for the creation and activation of an end-to-end network slice, including the creation and configuration of all the virtual components that are entailed in the slice. This metric takes into account the execution of two main steps in the neutral host platform workflow: the slice creation and activation (see Section 5.2).

- Slice Creation Time (SCT): refers to the amount of time it takes the Slice Manager to return the results of a submitted slice creation request to an end-user. This operation includes the sequential creation of all the chunks belonging to the slice and the grouping of those chunks. This time is measured from the moment when the creation request of a slice is sent to the Slice Manager, until receiving the confirmation that the slice was created.
- Slice Activation Time (SAT): refers to the amount of time it takes the Slice Manager to return the results of a submitted slice activation request to an end-user. This operation includes the instantiation of the mobile core and the configuration of the corresponding PLMNID in the RAN nodes included in the slice. This time is measured from the moment that the request is sent to the Slice Manager, until receiving the confirmation that the slice is ready to be used. Such confirmation is provided after receiving the acknowledgement from OpenStack about the mobile core instantiation and from the RAN Controller regarding the radio nodes configuration. Note that still additional seconds might be required to complete both operations as well as to finalize the Day1 configurations on the mobile core (based on cloud-init).

Henceforth, in the scope of the proposed framework, the SDT can be computed according to the following equation:

(1) SDT = SCT + SAT

To compute this KPI, a custom Python script was developed to automate the slice deployment, time measurement and slice removal by sending the required REST API calls to the platform Slice Manager. This script measures and stores the times involved in each operation in a database, simulating user requests (like the ones done via the Dashboard).

#### 6.1.4. Service Instantiation Time (SIT)

The time required for the provisioning and deployment of an NS over a given slice. This operation includes three main actions, namely:

- Set up of the networking in OpenStack required to connect each VNF included in the NS with the Monitoring component;
- Computation of the VNFs allocation (i.e., VNF-to-compute-chunk mapping) according to the algorithm employed by the Resource Placement component;
- Deployment and configuration of the NS instance through OSM as NFVO.

In essence, the SIT is measured from the instant when the instantiation request of an NS is sent to the Slice Manager, until the moment when the service instantiation is completed, i.e., when all the virtual components that are entailed in the service descriptor are active and running. Since in the proposed framework OSM is used as NFVO, the indication of a successful deployment is triggered when the service instance in OSM appears as running (operational status) and configured (configuration status).

As with the SDT, to compute this KPI, a custom Python script was used to automate the service instantiation over a given slice by sending the required REST API calls to the Slice Manager. For this test, all the required descriptors are available in the platform Catalogue and onboarded to OSM. Likewise, all the required images are already available in OpenStack, therefore only the instantiation time is considered without including the descriptors creation and onboarding processes.

#### 6.1.5. Service Scaling Time (SST)

The time required to launch an additional instance of a specific VNF contained in a given NS. This operation is requested via the platform Dashboard as a particular case of reaction in the face of an alarm triggering event. Once a reaction request for a given NS is launched, the rule associated with that event for horizontal scaling (i.e., scale-out/in) is retrieved and the corresponding scaling request is consequently delegated to OSM (manually triggered approach).

In particular, the SST is measured from the instant when the reaction request of an NS is sent to the Slice Manager, until the moment when the new instance is running. As with the SIT, a scaling request is successfully completed upon the appearance of the running indicator as operational status in OSM. The time required by the Monitoring component for anomaly detection and alarm triggering is not contemplated by this metric.

As with the previous two metrics, this KPI is measured via a custom Python script that automates the service scaling operation by sending the required REST API calls to the Slice Manager. After completing this action, the script also performs the service and slice removal to leave the system in the original state before repeating the entire sequence (i.e., slice deployment; service instantiation; service scaling; service removal; slice removal). Multiple iterations of this deployment lifecycle are conducted to obtain meaningful results.

### 6.2. Results Analysis

**Figure 5:**
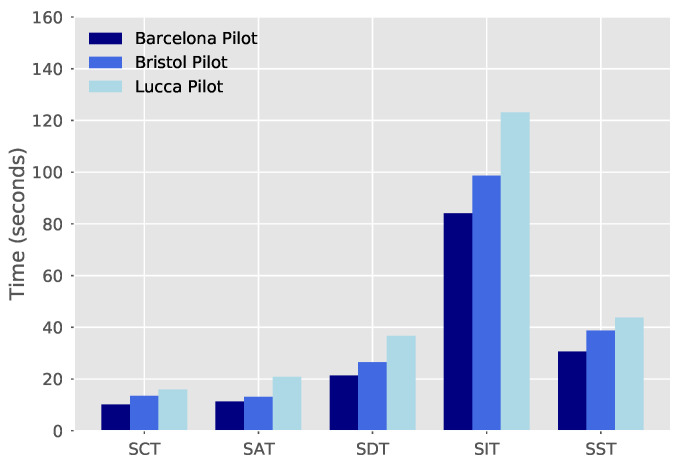
Deployment times of neutral host platform.

To demonstrate the benefits of the proposed framework, we deploy three slices over the neutral host infrastructure and measure the required SDT as expressed in Equation (1). The composition of such slices consists of one compute chunk, one network chunk and one radio chunk with radio nodes to provide cellular access to the end-users. Additionally, we deploy an NS with a “moderate” level of complexity (composed of four VNFs and two VLs) over such slices and, afterwards, we scale out one of the involved VNFs. The corresponding time performance results, averaged over 30 iterations, appear in Figure 5 considering each one of the involved operations over the three city pilots.

In Figure 5, we can appreciate that, in the considered scenarios, the slice deployment takes on average less than 37 s. For the sake of completeness, the associated SCT and SAT values are also included in the figure to better illustrate the impact of both operations on the resulting SDT. Meanwhile, the instantiation of the four VNFs composing the considered service is completed in around 84, 96, and 123 s over the Barcelona, Bristol and Lucca pilots, respectively. In terms of scaling, average SSTs of less than 45 s are also observed.

Although obtained results in most of the cases are very well aligned with the expected KPI outcomes, some differences in performance are observed when comparing the three city testbeds. In particular, higher SDT, SIT, and SST are experienced in the Lucca pilot, which is mainly due to the smaller capabilities of the servers deployed in that city. Nevertheless, observed differences between the three pilots are not significant, and in overall, this analysis demonstrates the good performance, in terms of deployment times, of the proposed neutral host solution to be deployed in city-wide 5G infrastructures.

**Figure 6:**
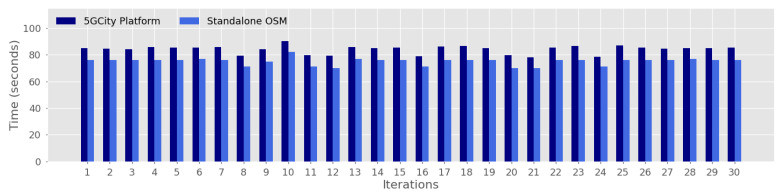
Time overhead against standalone OSM for Service Instantiation Time.

**Figure 7:**
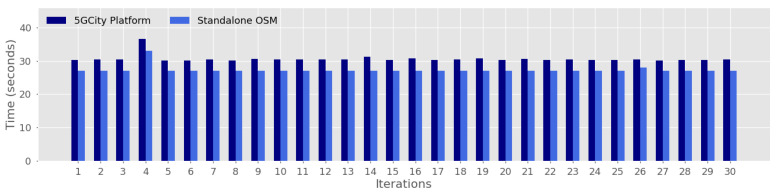
Time overhead against standalone OSM for Service Scaling Time.

To evaluate the impact of using the proposed framework for service orchestration, we compare the obtained results for SIT and SST considering as baseline the standalone use of OSM to perform the instantiation and scaling operations. Figure 6 and Figure 7 illustrate the performed comparison at a more granular level by depicting each one of the 30 iterations considered in this evaluation, over the Barcelona testbed. The motivation behind this analysis is to quantify the time overhead incurred by the proposed platform during the instantiation (Figure 6) and scaling (Figure 7) actions that are not directly introduced by the underlying NFVO.

Figure 6 shows that the overhead in terms of SIT remains acceptable, with values around 10 s in the performed evaluation. This overhead is due to the time required by the two first tasks listed before during the definition of the SIT KPI (i.e., networking setup and placement computation, see Section 6.1.4), which are performed by the proposed platform before conducting the third task, which directly corresponds to the service instantiation executed through OSM.

Likewise, Figure 7 evidences low overhead values in terms of SST, which are around 3 additional seconds. In this case, the observed overhead is a result of the automated reaction management that is done by the Slice Manager to retrieve the associated information (such as scaling type and identifier of the involved VNF) that is needed to handle this request, before actually conducting the service scaling via OSM.

Summing up, Table 1 compiles the average results obtained for each one of the five KPIs considered in this evaluation during the trials conducted in the three city pilots.

In addition to the previously discussed results in terms of SDT, SIT, and SST, the User Experienced Data Rate and Data Plane Delay measurements are also outlined in this table.

Regarding the User Experienced Data Rate, measured values report the cumulative DL throughput achieved by end-users in the considered multitenant scenario with three concurrent active slices, sampled every second during a period of 60 s. As for the Data Plane Delay, values included in Table 1 were measured against a remote server located in the edge computing hosts of each testbed. Therefore, obtained results show the network latency incurred between user equipment and edge computing instances, without including the processing time of network functions. These measurements may be impacted by several factors, such as the existing traffic load, distance from radio nodes, and propagation conditions.

Overall, the conducted trials and performed evaluations validate the feasibility of the proposed neutral host framework by demonstrating the correct operation and benefits of its technology components. Furthermore, regarding the slicing and orchestration (main research focus of our solution), related results demonstrate that the developed platform performs well, achieving the 5G PPP programmatic KPI for *Service Creation Times in minutes instead of hours*.

## 7. Conclusions

Turning a city into a distributed, multitenant and neutral host model-compliant infrastructure demands a comprehensive framework able to support and integrate end-to-end 5G services upon different network technologies. Towards such a goal, this article provides insights into the design and deployment of a three-tier infrastructure and an orchestration platform that enables municipalities and infrastructure providers to create dynamic end-to-end slices composed of both virtualized cloud/edge and network resources and to lease such slices to third party operators/verticals. The developed solution also provides lifecycle management and orchestration of 5G-based edge services, together with the control of the available underlying city-wide infrastructure. Through the execution of use case trials, the benefits of using the neutral host model for deploying, provisioning, and managing vertical services over a virtualized and shared infrastructure was demonstrated. Moreover, obtained results confirm the feasibility of the proposed framework to be deployed as a neutral host solution in city-wide 5G infrastructures.

Our future work will further exploit, in the context of the 5GVictori project, the potential of the 5GUK testbed to develop media vertical demos and experimentation scenarios over common large-scale field trials. In addition, we will work on the evolution of neutral host platform components, such as the Slice Manager and the RAN Controller, to support 5G Non-Standalone (NSA) and SA technologies together with the deployment of slices with distributed mobile core architectures based on the separation of user and control plane functions.

## Figures and Tables

**Table 1:** KPI measurements.

KPI	Barcelona	Bristol	Lucca
User Experienced Data Rate	44.7 Mbps	45.5 Mbps	44.7 Mbps
Data Plane Delay	9.85 ms	8.5 ms	8 ms
Slice Deployment Time	21.35 s	26.53 s	36.72 s
Service Instantiation Time	84.04 s	98.63 s	123.09 s
Service Scaling Time	30.59 s	38.74 s	43.68 s

## Data Availability

Not applicable.

## Abbreviations

The following abbreviations are used in this manuscript:

3GPP	3rd Generation Partnership Project
5G PPP	5G Public Private Partnership
5GNR	5G New Radio
AAA	Authentication, Authorization, and Accounting
C-RAN	Cloud Radio Access Network
CAPEX	Capital Expenditure
DC	Data Center
DHCP	Dynamic Host Configuration Protocol
DL	Down-Link
DNS	Domain Name System
eMBB	enhanced Mobile Broadband
ETSI	European Telecommunications Standards Institute
EU	European Union
GUI	Graphical User Interface
IMT	International Mobile Telecommunication
ITU	International Telecommunication Union
KPI	Key Performance Indicator
LoRa	Long Range
LTE	Long Term Evolution
MANO	Management and Orchestration
MEAO	MEC Application Orchestrator
MEC	Multi-Access Edge Computing
mMTC	massive Machine Type Communications
MOCN	Multi-Operator Core Network
MORAN	Multi-Operator Radio Access Network
MVNO	Mobile Virtual Network Operator
NFV	Network Function Virtualization
NFVI	Network Functions Virtualization Infrastructure
NFVO	NFV Orchestrator
NGMN	Next GenerationMobile Networks
NS	Network Service
NSA	Non-Standalone
OSM	Open Source MANO
PLMNID	Public Land Mobile Network ID
RAN	Radio Access Network
RAT	Radio Access Technologies
ROI	Return of Investments
SDK	Software Development Kit
SDN	Software-Defined Networks
SDR	Software Defined Radio
SLA	Service Level Agreement
UE	User Equipment
UL	Up-Link
URLLC	Ultra-Reliable Low Latency Communications
vEPC	virtual Evolved Packet Core
VIM	Virtual Infrastructure Manager
VLAN	Virtual Local Area Network
VM	Virtual Machine
VNF	Virtual Network Function
VPN	Virtual Private Network
